# A review of sexual misconduct in dentistry

**DOI:** 10.1038/s41415-025-8811-3

**Published:** 2025-09-26

**Authors:** Aaron Drovandi, Gabrielle M. Finn

**Affiliations:** Division of Medical Education, School of Medical Sciences, University of Manchester, Manchester, United Kingdom

## Abstract

**Background** Sexual misconduct in dentistry includes inappropriate behaviours from patients or staff, such as unwanted advances, comments, gestures, or physical contact. This review aimed to explore the literature for the prevalence, causes, impacts and interventions of sexual misconduct in dentistry, to identify gaps and inform policy development and future research.

**Methods** A systematic search of the academic literature was conducted to identify eligible articles published between 1 January 2010 and 31 October 2024. Eligible articles were those of any study design that captured and reported on data relating to sexual misconduct in dental care settings and were available in the English language. Quantitative data were summarised narratively and key findings grouped according to the common themes identified.

**Results** A total of 2,238 articles were screened, with 23 peer-reviewed articles meeting the inclusion criteria. Findings revealed a limited global representation and general restriction to sexual misconduct prevalence data, with no studies reporting on the implementation or effectiveness of interventions targeting sexual misconduct behaviours. Prevalence data were broad (5-86%), with underreporting of incidences also highlighted and witnessing sexual misconduct also common (25-40% within studies). Contributing factors to sexual misconduct included power hierarchies, informal settings involving alcohol, poor reporting mechanisms and fear of retaliation. Male dentists and patients were overrepresented as perpetrators. Victims experienced emotional trauma, professional disengagement and reluctance to report. Recommendations from studies emphasised training, robust policies, supportive environments, restorative justice and balanced media representation.

**Conclusion** Systemic change is urgently needed to address sexual misconduct in dentistry. Limited UK-based research indicates the need to prioritise qualitative insights and intervention effectiveness to create safer, equitable dental workplaces.

**Supplementary Information:**

Zusatzmaterial online: Zu diesem Beitrag sind unter 10.1038/s41415-025-8811-3 für autorisierte Leser zusätzliche Dateien abrufbar.

## Key points

- Sexual misconduct encompasses a range of inappropriate behaviours, though little data are available on the prevalence and causes of sexual misconduct in dental settings.
- There were no studies identified in this review which included data on dental patient experiences of sexual misconduct, and no studies which investigated interventions targeted at preventing or managing instances of sexual misconduct.
- Experiences of and witnessing sexual misconduct in dental settings were highest among dental hygienists compared to dental students and dentists, with unclear reporting mechanisms and lack of trust in existing reporting systems.

**Supplementary Information:**

Zusatzmaterial online: Zu diesem Beitrag sind unter 10.1038/s41415-025-8811-3 für autorisierte Leser zusätzliche Dateien abrufbar.

## Introduction

Sexual misconduct is a broad term encompassing a range of behaviours, subject to differences in interpretation according to the setting. Within the United Kingdom (UK), the Equality Act 2010 as interpreted by the British Medical Association (BMA) states that ‘someone sexually harasses another person in employment if they: 1) engage in unwanted conduct of a sexual nature; and 2) the conduct has the purpose or effect of either violating the other person's dignity or creating an intimidating, hostile, degrading, humiliating or offensive environment for them'.^1^^,^^2^ It is noted that such behaviours can occur in person or electronically (via phone, text, email, or online). Certain forms of sexual misconduct, such as stalking, incident exposure and ‘upskirting', are criminal offences and amount to sexual assault. The UK Worker Protection Act 2023, an amendment of the Equality Act 2010, requires employers by law to take ‘reasonable steps' to prevent sexual misconduct and create a safe working environment.^3^

Within the dental workplace setting, sexual misconduct might involve a range of inappropriate behaviours, including unwelcome sexual advances, comments, gestures and physical contact that are of a sexual nature and not relevant to patient care.^4^ These behaviours can occur between several parties, including dental practitioners, dental office staff, patients, their family members or carers, students, or educators.^5^ Incidences of sexual misconduct may lead to hostile and unsafe environments and cause lasting damage to the wellbeing of victims, as well as the reputation of professionals and their profession.^5^^,^^6^ Factors influencing these behaviours include imbalanced power dynamics, a lack of clear policies on misconduct, inadequate training on managing misconduct, as well as unclear or insufficient reporting mechanisms and a tolerance of inappropriate behaviours emboldening offenders.^7^^,^^8^^,^^9^

Despite the creation of social movements, such as #MeToo, increasing societal focus on gender-based discrimination, and reports about sexism and sexual misconduct in the medical field,^10^ there is limited literature on sexual misconduct in dentistry. This review aims to identify and analyse published evidence about sexual misconduct in dental working environments.

## Methods

This rapid review was conducted according to elements of the Preferred Reporting Items for Systematic Reviews and Meta-Analyses (PRISMA) guidelines.^11^ For the purposes of this review, ‘sexual misconduct' was defined according to the University of Law's definition, encompassing actions or behaviours that include sexual violence, assault, harassment, bullying or any form of sexual victimisation.^12^

### Search strategy

In total, 30 databases were searched for studies which reported on sexual misconduct prevalence, causes, impacts and interventions for sexual misconduct in dentistry workplace settings (see Appendix 1 for the full database list). Searches were conducted in the English language only, and captured articles published between 1 January 2010 and 31 October 2024. The search terms and search strategy were developed between both authors and an information search specialist from the University of Manchester, and agreed to by the General Dental Council (GDC) (UK), who sponsored this research. The search string combined three term groups using truncation symbols and Boolean operators ‘AND' and ‘OR' within ‘Title' and ‘Abstract' fields. The term groups included: 1) sex terms (e.g., sexual); 2) misconduct terms (e.g., assault); and 3) dental terms (e.g., dentistry) (see Appendix 2 for the full list of terms used in the search). Reference and citation lists of eligible studies and relevant systematic reviews were manually searched to identify additional articles not captured by the search strategy.

### Study selection

The eligibility criteria are shown in Table 1. In brief, eligible articles were those that captured and reported on sexual misconduct within dental workplace and training settings, including prevalence, causes, consequences and interventions targeted at sexual misconduct. Searches were conducted by both authors and a third academic (MB; see acknowledgements), with search results imported into EndNote for title and abstract screening. Potentially eligible articles underwent full-text review with all three academics agreeing on final inclusions.

**Table 1:** Inclusion and exclusion criteria for the systematic search

**Inclusion**	**Exclusion**
**Population** Studies involving dental professionals (dentists, dental care professionals, dental technicians, dental nurses), dental students, and patients in dental care settings	Studies that included dental professionals though data were mixed with other health professions and did not explicitly state outcomes specific to dental professionals
**Phenomenon of interest** Studies capturing and reporting on the prevalence of sexual misconduct within the context of dentistry, including details of perpetrator and victim. Also studies that reported on policies or interventions targeted at preventing or managing instances of sexual misconduct	Studies that did not provide empirical data on sexual misconduct in dental settings or with dental professionals
**Types of studies** All study designs (e.g., quantitative, qualitative, mixed methods)	Literature that is solely editorial or opinion-based without empirical data or case analysis
**Language** Studies in English and other languages, provided an English abstract or translation is available	N/A
**Timeframe** January 2010 - October 2024	N/A

### Data extraction and analysis

A shared excel workbook was used to manage included articles, with data extracted including: title; authors; year published; study design; country; setting; funding; population; sample size; participant inclusion and exclusion criteria; definition and description of sexual misconduct; data collection methods; key findings; author conclusions; and implications for practice and further research. Data extraction was conducted by the three researchers and a random selection of articles (n = 5) were quality-checked by a second reviewer. Quantitative data were summarised descriptively and key findings grouped according to common themes identified by the researchers. Due to the small sample size of papers included, thematic analysis was conducted within the excel workbook. The funder requirements were for a rapid evidence synthesis; therefore, a quality appraisal was not performed on the eligible articles.

## Results

**Fig. 1:**
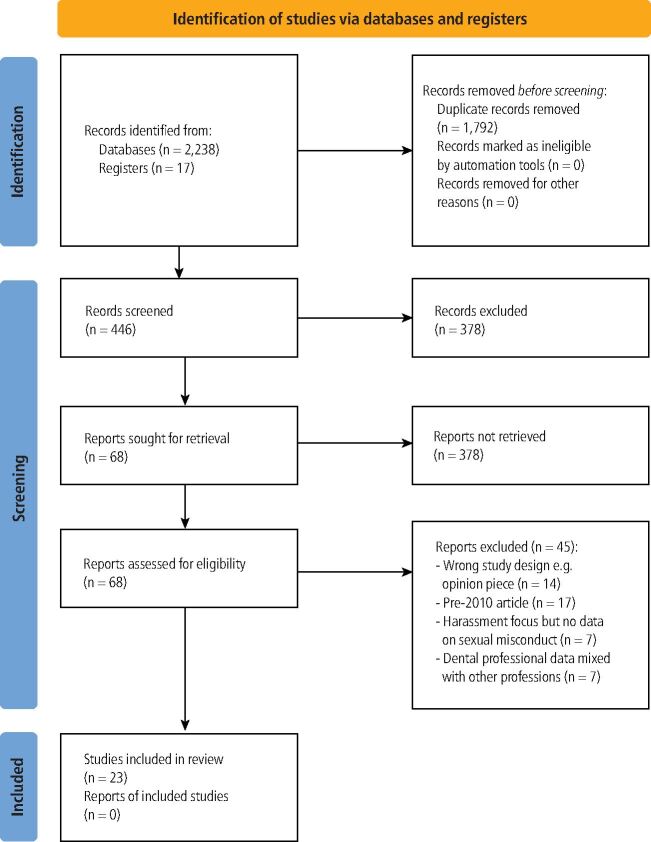
PRISMA flow diagram of the systematic search results

The search identified 2,238 studies; after removing duplicates, 446 studies underwent title and abstract screening, with 378 of these excluded. The remaining 68 studies underwent full-text assessment, with 45 excluded due to study design, lack of relevant outcome data, wrong participant population, or year of publication. Figure 1 illustrates the searching and screening process, with 23 peer-reviewed published studies from 2010 to current included. ^13^^,^^14^^,^^15^^,^^16^^,^^17^^,^^18^^,^^19^^,^^20^^,^^21^^,^^22^^,^^23^^,^^24^^,^^25^^,^^26^^,^^27^^,^^28^^,^^29^^,^^30^^,^^31^^,^^32^^,^^33^^,^^34^^,^^35^ Online Supplementary Table 1 provides a summary of key extracted data. Papers used terminology of ‘sexual misconduct' and ‘sexual harassment'. From herein, we refer to ‘sexual misconduct' within our discussion.

### Defining sexual misconduct

Definitions were variously described; eight papers did not define terms.^15^^,^^16^^,^^17^^,^^21^^,^^24^^,^^31^^,^^32^^,^^34^ Definitions from established organisations, such as the World Health Organization or the National Academies of Sciences, Engineering, and Medicine 2018 report, were cited. Synthesising the various definitions provided the following: sexual misconduct encompasses a range of unwelcome behaviours, including gender harassment, unwanted sexual attention, and sexual coercion. It may involve verbal, non-verbal, or physical actions that create a hostile or intimidating environment, interfering with an individual's work performance.

### Study characteristics

Of the 23 eligible studies, 17 reported on cross-sectional surveys mainly focused on perceptions and experiences of sexual misconduct among participants, including witnessing harassment. Of these, four focused on dental students,^20^^,^^25^^,^^27^^,^^31^ six on dental hygienists,^16^^,^^21^^,^^23^^,^^26^^,^^30^^,^^35^ two on dental surgeons,^33^^,^^34^ and five on a range of dental professionals (two including students).^13^^,^^14^^,^^15^^,^^22^^,^^24^ The remaining six studies analysed harassment case proceedings (n = 2),^18^^,^^19^ tribunal data (n = 1),^29^ media releases (n = 1),^17^ or had a mixed-methods approach (n = 2).^28^^,^^32^ Nine studies recruited participants from the United States (USA), three from the UK, three from Canada, two from South Korea, one from Brazil, one from New Zealand, one from Australia, one from Nigeria, one from Pakistan, and one from four countries (USA, Bulgaria, Brazil, India). From these 23 studies, there was a total participant population of 8,988 dental students and professionals, and data from 794 tribunal decisions, 362 cases and 122 media articles.

### Experiences of sexual misconduct

#### Patients

None of the studies reported on patients as victims of sexual misconduct in dental settings.

#### Dental students

Seven survey studies included dental students reporting on their experiences as victims of sexual harassment;^13^^,^^15^^,^^20^^,^^25^^,^^27^^,^^31^^,^^32^ although, two studies combined student data with that of academic staff or dental professionals.^13^^,^^15^ These five remaining studies found that between 5% and 22% of participating students had experienced sexual harassment. Of these, two reported that male students were more likely to be victims of sexual harassment compared to female students,^20^^,^^31^ while two others reported the opposite,^13^^,^^17^^,^^27^^,^^32^ and others did not specify. Types of harassment experienced by students included sexually implicit jokes or stories, being asked on a date, uncomfortable touching, staring/leering, sexual remarks/objectification, or sexual advances.

#### Dental hygienists

Six survey studies included dental hygienists reporting experiences of sexual misconduct, with prevalence ranging from approximately 25% (clearly stated in three studies)^17^^,^^21^^,^^23^ up to 86% in one study.^30^ Types of harassment were similar to dental students, such as sexually implicit jokes or stories, sexual or crude remarks, uncomfortable touching, and asking for dates. Some also were shown pornographic materials, or had patients expose their genitals or masturbate in front of them. Kim (2017) reported that 48.7% of female dental hygienists surveyed had experienced sexual harassment in their workplace.^26^ From the survey items, ‘I have heard a misogynistic comment or been the target of someone's abusive language' had the highest response rate (23.2%), followed by ‘I have felt upset or disturbed because I suspected someone treated me as a sexual object by assessing my look, clothing, body, etc.,' (17.0%). Verbal sexual harassment was experienced more commonly (73.2%) than physical harassment.

#### Dentists and dental surgeons

Five survey studies included dentists or dental surgeons reporting their experiences relating to sexual harassment,^14^^,^^22^^,^^24^^,^^33^^,^^34^ with prevalence lower than for dental students and hygienists, with two studies reporting 7% of participants had experienced harassment,^14^^,^^34^ one reporting 21%,^22^ and one reporting 29%.^33^ One study did not clearly report prevalence. Types of harassment were the same as those experienced by dental students and hygienists.^24^

### Witnessing sexual misconduct

Of the few studies that reported on witnessing sexual misconduct, the prevalence was generally the same or slightly higher than experiencing sexual misconduct. One study reported that 25% of dental students had witnessed a fellow student being sexually harassed,^20^ while another reported that 40% of participants had witnessed sexual harassment of their colleagues.^15^ One study reported that three-quarters of those who witnessed a colleague being sexually harassed did not intervene.^20^

### Perpetrators of sexual misconduct

Ten studies reported on the characteristics of perpetrators,^15^^,^^20^^,^^22^^,^^24^^,^^27^^,^^29^^,^^30^^,^^31^^,^^35^ with patients and colleagues (especially those senior to the victim) being the two most frequent perpetrators, and for dental students, professors/academics were also frequently cited, with men being disproportionately involved as perpetrators. For example, Millbank *et al.*, (2020) found that 80.4% of misconduct perpetrators were male, and specifically for inappropriate sexual contact, 96.7% of cases involved men.^29^ Patel (2021) reported on sexual harassment by patients, with quotes such as ‘most patients with inappropriate sexual behaviours were men over 60' and ‘patient was a man who refused to make an appointment until he was added on my Facebook page and could contact me directly - stalking-type behaviour' providing examples for this trend.^30^ In addition to gender, dentists were overrepresented relative to their workforce proportion, with Kim (2017) reporting dentists were the most frequent offenders (67.3%).^26^

### Settings

For dental students, sexual harassment mostly occurred within dental school, followed by in dental clinics (assumedly as part of placement),^13^^,^^20^^,^^25^^,^^31^ and one study also reporting that students experienced harassment over the phone.^20^ For dental hygienists, dentists and dental surgeons, incidents occurred within dental clinics, most of which were during normal working hours.^14^^,^^18^^,^^23^^,^^26^ However, some participants reported that harassment took place outside of normal working hours or outside of the workplace. Social gatherings, such as Christmas parties, was also raised in one of the tribunal cases,^19^ with other studies reporting on case data also highlight digital spaces as being an avenue for harassment, such as social media platforms.^28^ Heaton *et al.*, (2020) found that conferences and networking events in particular were common environments in which sexual misconduct occurred.^22^

### Contributing factors

Heaton *et al.*, (2020) posited that cultural dynamics, informal networking settings and hierarchical power structures were significant facilitators of harassment.^22^ Further, alcohol consumption at conferences and networking events was noted as a contributing factor to inappropriate behaviour. One study's participants believed instances of harassment among students was often partially the fault of the student experiencing harassment, for not exhibiting appropriate behaviours themselves, or due to pre-existing relationships to the perpetrator.^20^

Ellis and Johnson (2020) concluded that newspapers often focus on and sensationalise cases of unprofessional behaviour.^17^ This sensationalism may cause unnecessary concern and additional anxiety among patients, as media articles of dental professional behaviours focus on a small number of extreme cases involving incidents of significant harm to patients or the public, raising public concerns about the profession. Reporting of dental professional behaviours juxtaposes issues of crime, immoral behaviour and dishonesty with dentists' professional standing. Cases reported in the news are often sensationalist, unusual and do not appear to reflect fitness to practise (FtP) cases heard by the GDC.^17^

### Responses and reporting

A common theme across the literature was that of uncertainty of reporting pathways available to registrants, for example, Garbin *et al.*, (2010) reported that nearly half of participants would not know what to do if they were harassed.^20^ Relating to this, there was a strong trend of victims of sexual harassment not reporting and trying to simply ignore perpetrators, which may (in addition to not being aware of reporting options available) be also due to there being discomfort associated with reporting someone, or a lack of trust that any action would be taken: ‘if I am grabbed I immediately let my doctor know and he handles it with dismissal [of my concerns]'.^30^ Investigating other reasons behind not reporting were limited, with two studies reporting that hygienists and students raised that they were fearful of retaliation from reporting and losing their employment (or place at university).^21^^,^^25^

Several studies reported that participants were successfully able to distract patients after remarks/advances were made, with one study stating that this approach led to satisfactory outcomes in half of instances.^30^ Examples of this include Kim (2017) reporting that 36.4% of victims took no specific action.^26^ Al-Jewair *et al.*, (2024) reported that victims often adopted passive responses, such as ignoring or avoiding the situation.^13^ For those that did report, several participants from studies indicated that they were simply ignored or mocked and told ‘it is the way it is': ‘I always told the office manager and doctor and assistant about his behaviour, and it was mostly laughed off as in “that's just how he is”'.^22^^,^^27^^,^^30^

### Consequences of sexual misconduct

**Fig. 2:**
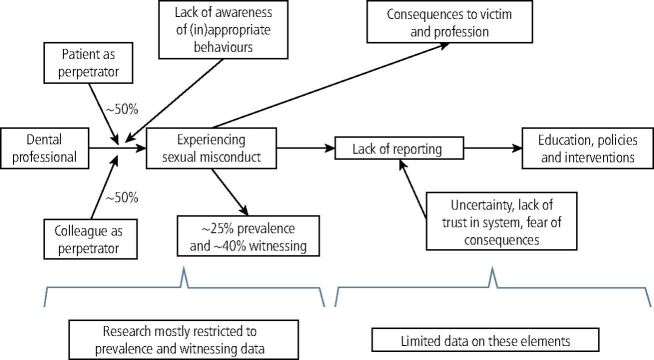
Illustration of the key findings of the review

Dental students reported that being a victim of sexual harassment had a negative impact on their academic performance, reduced their enthusiasm for studying and working in dental practice, and caused recurrent upsetting memories surrounding the incident.^24^^,^^31^ Dental hygienists also found harassment affected their enthusiasm for working, strained relationships with patients, caused a negative impact on clinical performance and created a negative work culture, with stress and psychological distress.^21^ Figure 2 highlights the key elements found from the review, including victim, perpetrator, consequences, reporting and required interventions. Most published data are restricted to prevalence of sexual misconduct, including information on perpetrators and how it affects victims. Comparatively, there is little data on how victims report (if they report) on sexual misconduct, with most studies highlighting the need for improved health professional education on acceptable and unacceptable behaviours, creating safe working spaces, and policies and interventions targeted at reducing the occurrence of harassment, as well as clearer pathways for reporting.

### Interventions

None of the studies implemented and evaluated interventions to address sexual misconduct. Only one study by Al-Jewair *et al.*, (2024) provided a potential framework - the Intervene, Report, Document, Access support, Resolve (IRDAR) framework - suggesting that clear protocols such as IRDAR may help address harassment effectively.^13^

### Recommendations for future research

A thematic analysis of the recommendations for future research highlighted the need to explore several areas. Firstly, systemic and cultural factors influencing sexual misconduct experiences and reporting. Literature suggested investigating systemic factors and broader demographic representation influencing harassment prevalence and perceptions. In addition, there was a call to examine cultural and institutional influences on harassment in dental education and professional environments; a global lens was recommended. Secondly, through qualitative methodologies, analyse decision-making processes for misconduct cases, with the aim of understanding factors driving variations in outcomes for different registrants. Thirdly, expand research to broader settings beyond limited cohorts, as well as incorporate more qualitative methodologies, such as interviews and focus groups, to understand the psychological and social impact of harassment on students and professionals. This could also include investigating the effectiveness of reporting mechanisms and their alignment with organisational policies. Finally, as much of the literature was from the USA, suggestions for future work focused on the evaluation of restorative justice practices, including to other professional sectors to promote cultural change, include, for example, evaluating the long-term impacts of programmes of restorative justice and investigating trends in workplace violence.

## Discussion

### Summary of findings

This report examined the literature relating to sexual misconduct within dentistry. There was limited literature, with most of what was available published from USA data. In sum, sexual misconduct is significantly under-reported in dentistry, with prevalence ranging from 5-48% depending on population and study context. Verbal harassment was the most frequent form of sexual misconduct reported, followed by inappropriate physical contact and visual harassment. Our analysis identified several contributing factors, including but not limited to: workplace hierarchical power dynamics; informal workplace cultures, particularly during events involving alcohol; and a lack of clear reporting mechanisms; and fear of retaliation. The main barriers to reporting were that many victims cited unclear reporting pathways, fear of reprisal, and lack of trust in administrative support as reasons for not reporting incidents. Male professionals were disproportionately represented as perpetrators, with dentists frequently implicated. Victims experience emotional trauma, professional disengagement and reluctance to report, and organisations risk of reputation damage, patient loss, decline in staff morale and legal consequences. There is also an overall risk for the dental sector in terms of a decline in public trust and professional standing.

This review found no studies which trialled interventions. However, several recommendations for interventions were identified, including educational programmes, structured reporting frameworks and restorative justice practices. There is a need to develop robust policies and training to prevent and address misconduct, as well as promoting supportive organisational cultures. Somewhat USA-centrically, there was the suggestion of implementing restorative justice measures to rebuild trust and improve workplace dynamics. There is also a need to advocate for balanced media reporting to mitigate sensationalism and protect the profession's reputation and retain public confidence.

Finally, a number of evidence gaps were identified, including the limited global and cultural representation in studies, as well as the lack of longitudinal research on the effectiveness of interventions. Furthermore, there was insufficient exploration of patient experiences and the impact on care delivery.

### Evidence from other sectors

Within the field of medicine, key organisations, such as the BMA and the Royal College of Surgeons of England, have lobbied the government and NHS (National Health Service) organisations regarding the working party recommendations and the need for reforms of reporting and investigation processes of sexual misconduct.^36^ There is significantly more research pertaining to sexual misconduct in medicine. While much will be relevant and transferrable to dentistry, there is a need to evidence the dental-specific context as the environment, working practices and cultures differ.

For example, a 2024 report by the BMA reported on a survey of 2,458 doctors conducted in 2021, finding that 31% of female and 23% of male respondents experienced unwanted physical conduct in their workplace.^2^ Further, 56% of female and 28% of male respondents received unwanted verbal conduct related to their gender, and 42% of all respondents who witnessed or experienced an issue relating to sexism felt they couldn't report it.^2^ Similarly, a paper from Begeny *et al.*, (2023) concluded that, ‘sexual misconduct in the past five years has been experienced widely, with women affected disproportionately. Accountable organisations are not regarded as dealing adequately with this issue'.^37^

### Patient experiences

While our review did not identify any studies that considered patient experience of sexual misconduct, the wider healthcare literature delineates important considerations with respect to patient experiences. Thurston *et al.*, (2019) detailed how sexual harassment has health consequences for those affected and is associated with poorer physical and mental health.^36^^,^^38^ A study in Germany considering the professional sexual misconduct towards patients indicated a high proportion of sexual contact before the age of 18.^39^ Such experience of sexual abuse in childhood and adolescence can have profound influence on the rest of a patient's life. Consequences can include several physical illnesses,^40^ psychological problems and social impairments.^41^ Professional sexual misconduct may significantly violate trust in healthcare professionals and institutions, with possibly additional harmful consequences for the health of affected subjects due to avoidance and non-compliance.^39^ Bloom *et al.*, (1999) state emphatically that it is never acceptable to blame the patient for the sexual misconduct.^42^ Despite being relatively rare, sexual misconduct significantly undermines public trust and can cause severe psychological trauma to patients, as well as hesitation in seeking future medical care.^43^ It is also posited that patients are reluctant to report due to the prospect of reliving the trauma,^43^ due to fear of not being believed, uncertainty about what constitutes misconduct, and the power imbalance in the physician-patient relationship.^44^ Physicians and colleagues may also fail to report misconduct due to fear of retaliation, assumptions that others will act, or lack of mandatory reporting requirements.^44^ Literature from medicine advocates for clinics adopting policies that emphasise consent, respect and sensitivity to patients' histories, including trauma-informed care.^43^

### Considerations for professional standards and fitness to practise

Overall, this review found a lack of clarity regarding how to report incidents of sexual misconduct; we recommend regulators and dental sector organisations co-develop explicit standards with respect to sexual misconduct to protect registrants and the public. Currently, sexual misconduct is not specifically listed within the standards for GDC registrants,^45^ though is tangentially referred to in Standard 9 which discusses personal behaviour, and Standard 8 which discusses referrals based on ‘indecency'. The GDC FtP guidelines stipulate erasure from the register is possible when behaviours are fundamentally incompatible with being a dental professional, with convictions or findings of a sexual nature, including involvement in any form of child pornography contributing to such a conclusion.^46^ However, professional standards documentation is still evolving, with more recent GDC Interim Orders Committee guidelines providing more specific guidance relating to sexual misconduct, including terminology and impact of repeat offenses.^47^

In a recent report regarding the role of remediation in FtP within dentistry, consideration was given to sexual misconduct cases.^48^ The healthcare regulators interviewed in the study reported being ‘hawkish' with respect to behavioural and attitudinal complaints, particularly those relating to sexual misconduct. There were notable differences with respect to whether regulators viewed sexual misconduct issues as being in scope for remediation. Concerning such sexual misconduct, it was reported that remediation might be difficult to demonstrate. However, it was suggested that sincere reflection and insight might provide assurance that a registrant had understood the impact of their actions and undertaken to behave differently in the future.

Finn and colleagues reported on the negative impact of FtP on the mental health of all involved.^49^ There is a cumulative negative impact on mental health, regardless of whether practise is found to be impaired. The complexity of the processes and a perceived lack of clarity on how decisions were reached often resulted in feelings of mistrust and unfairness; this will undoubtedly by exacerbated for cases of a sexual nature. Significant levels of stress and anxiety were created by the process, at times hindering the progress of investigations, as participants disengaged or left the profession. Dental professionals reported feeling professionally ostracised, like their professionalism was being called into question, or that they had been presumed ‘guilty' until they had proved otherwise. Such feelings often exacerbated feelings of stress and anxiety. Given the findings of Finn *et al.*, and the aforementioned trauma, sensationalism, and embarrassment reported to be associated with sexual misconduct cases in the included studies in this review, even greater care should be taken to signpost all parties to appropriate support services.^49^

### Gender and profession-related findings

The studies included highlighted a significant gender differential in terms of experiences of sexual misconduct. Women were more likely to be the victims and perpetrators were predominantly men. Dental hygienists represented the highest proportion of victims in the studies included, which has significant implications for the UK workforce where over 90% of dental hygienists are women. Furthermore, women make up 77% of the UK dental workforce, including 50% of dentists and 92% of all dental care professionals.^50^ There is need to ensure that all patients and healthcare professionals have safe environments in which to access healthcare or work; however, these data suggest that there must be a concerted effort to ensure women are aware of how to report issues and access support, especially in light of the concerns surrounding under-reporting. When considering the gender-related data, caution must be aired regarding making heteronormative assumptions, including about the sex and gender of both the perpetrator and the victim.

### Recommendations

The papers included presented a limited number of recommendations. Ellis and Johnson (2020) advocated for dental professional bodies to campaign for more balanced coverage of dentists and dental professionalism within news media, and continue to promote the good work of the profession.^17^ Kim (2017) suggested that sexual harassment should no longer be recognised solely as a personal problem, but also as a problem for the entire organisation and society.^26^ There were recommendations to implement preventive education programmes, establish stronger legal protections and enforcement, as well as develop supportive organisational policies to address incidents assertively. Finally, Al-Jewair *et al.* (2024) noted the importance of ensuring that all individuals have access to reporting mechanisms that they perceive as safe, effective and supportive.^13^

Beyond this review, there are articles related to state licensing boards in the USA that offer recommendations related to sexual misconduct by physicians.^51^^,^^52^ Key points include recognition that the spectrum of sexual misconduct ranges from grooming behaviours to assault, necessitating vigilance against even minor infractions to prevent escalation. Physician-patient relationships are inherently power-imbalanced and thus require strict ethical standards to protect vulnerable patients. With respect to regulation, while serious misconduct like assault may lead to immediate suspension or revocation, lesser infractions could involve education, monitoring, or behavioural treatment for re-entry into practice. Medical literature strongly advocates for trauma-informed investigations.

To address sexual misconduct in dentistry effectively, we propose that several key areas require attention, including training, the establishment of a safe workplace, clear reporting pathways, managing barriers to reporting, comprehensive policies, and raising patient awareness. Such strategies require the efforts of both dental sector organisations and members of the profession.

Training is essential for fostering a professional and respectful environment. Mandatory training should be implemented for all dental practitioners, staff and management, focusing on recognising, preventing and addressing sexual misconduct within dental practices. This training should emphasise ethical patient interactions, professional boundaries, consent and cultural sensitivity. Scenario-based learning, tailored specifically to dentistry, should be included. Supervisors and practice managers should receive specialised training to handle and investigate complaints effectively. Additionally, bystander intervention training should be introduced to empower all staff to recognise warning signs and intervene appropriately when witnessing or suspecting inappropriate behaviour.

Creating a safe workplace or learning environment is crucial to ensuring the wellbeing of patients, employees and students. Codes of conduct should be established, emphasising respect, professionalism and zero tolerance for sexual misconduct. Furthermore, fostering a supportive workplace culture where employees, students and patients feel secure in discussing concerns without fear of retaliation is vital.

Clear and accessible reporting pathways are fundamental to managing and addressing instances of sexual misconduct. A multichannel reporting system should be established, offering different routes to report concerns. This could include anonymous online tools, confidential contact points within the practice, and external reporting hotlines for cases requiring escalation. Procedures for reporting misconduct should be publicly communicated, with information displayed in prominent positions in relevant environments. The role of the regulator should be clearly communicated to manage expectations.

Policies play a critical role in addressing and managing issues of sexual misconduct. Comprehensive policies tailored to dentistry should be developed, clearly defining sexual misconduct, providing examples, and detailing reporting protocols and disciplinary procedures. Incident management guidelines should standardise the documentation and reporting process, ensuring that every complaint is formally logged and addressed. A zero-tolerance approach should be enforced, with clear consequences for any substantiated cases of misconduct. Policies must also comply with local legal and regulatory requirements and be periodically reviewed to incorporate updates or address gaps identified through feedback or incident reviews.

Despite no patient data being reported in this review, raising patient awareness remains an important consideration. Practices should display educational materials in waiting areas that outline patients' rights, acceptable professional conduct and how to report concerns. These materials should use clear and accessible language to accommodate diverse patient demographics. Statements about sexual misconduct policies could also be included in patient intake forms or consent documents, ensuring patients are informed of their rights. Tailored resources for vulnerable patient groups, such as minors, older patients, or individuals under sedation, should be provided to empower them to recognise inappropriate behaviour and report concerns.

To ensure these measures are effective, regular audits and evaluations should be conducted to assess training participation, policy compliance, and the effectiveness of reporting pathways. External oversight from regulators, professional bodies, or independent organisations could provide valuable input, particularly for sensitive cases requiring impartial investigation. Transparency is essential and practices should consider publishing annual summaries of improvements made, cases managed, and lessons learned, anonymised to protect confidentiality and to build trust and demonstrate accountability.

By addressing these areas, a safer environment for all parties can be created while ensuring robust systems are in place to prevent and manage incidents of sexual misconduct effectively.

### Strengths and limitations

When interpreting the findings of this article, readers should consider its strengths and limitations. This review used a comprehensive database search of the published literature to provide a reliable overview of current knowledge of sexual misconduct in dentistry, including consideration of a broad range of factors, such as reporting, consequences and contributing factors. However, the restriction of most included articles to cross-sectional surveys and the omission of patient experiences provide limited insights into a holistic understanding of sexual misconduct in dentistry. The lack of quality appraisal of these included studies may also limit reliability of the findings, though is of less concern compared to interventional studies.

## Conclusions

This review highlights the need for systemic changes in dentistry to address sexual misconduct effectively. A combined effort from regulators, educational institutions and professional bodies is critical to foster safer, more equitable environments in dental workplaces. The literature is limited to mostly USA-centric studies and those reporting prevalence data, with a paucity of studies piloting interventions. Future research should prioritise global perspectives, qualitative insights and evaluation of innovative interventions to combat this pervasive issue.

**Table:** Appendix 1 Databases searched using the search strategy

**Medical and health sciences databases**	**Regional and discipline-specific databases**
PsycINFO	Dentistry & Oral Sciences Source
PubMed	ASSIA
Embase	African Journals Online (AJOL)
CINAHL	Latin American and Caribbean Health Sciences Literature (LILACS)
Web of Science	Scientific Electronic Library Online (SciELO)
Cochrane Library	African Index Medicus (AIM)
Medline	KoreaMed
Scopus	Western Pacific Region Index Medicus (WPRIM)
AMED	China National Knowledge Infrastructure (CNKI)
Ovid Technologies	ThaiJO
ProQuest	e-Marefa
Academic Medicine	Philippine E-Journals (PEJ)
Google Scholar	J-STAGE
MedEdPORTAL	SA ePublications
BioMed Central	
Cochrane Collection Plus

**Table:** Appendix 2 The three term groups used in the search string and terms used within each group

**Sexual terms**	**Misconduct terms**	**Dental terms**
AND		
Sexual	Misconduct	Dent*
Sex*	Violence	Dental
	Attack	Dentistry
	Assault	
	Harassment	
	Bullying	
	Victimisation	

## Supplementary Information

Supplementary Table 1 (PDF 140KB)

## Data Availability

Data are available from the corresponding author on reasonable request.
