# Supplementary material for: A review of sexual misconduct in dentistry
Source: Br Dent J. 2025 Sep 26;239(6):393–401. doi: 10.1038/s41415-025-8811-3 (PMC12474545; doi:10.1038/s41415-025-8811-3)
Supplement: Supplementary file 1 — Supplementary Table 1 (PDF 140KB) [file 41415_2025_8811_MOESM1_ESM.pdf]

SI Table 1 Key data extracted from each eligible study

Study	Study Design	Country & Setting	Population Studied & Sample Size	Reported Aims/ Objectives	Data Collection Methods	Key Findings, and Notable Quotes, Excerpts or Observations
**Al-Jewair (2024)**	Cross-sectional survey and protocol development	United States; single university dental school	116 dental faculty and 156 dental students	Analyse factors contributing to workplace violence (WPV) and develop a prevention and management protocol	27-item survey on perceptions of WPV and institutional attention to WPV. OSHA WPV assessments.	Sexual harassment was not quantified separately to other forms of harassment, with verbal aggression most common. Developed a protocol for inappropriate patient behaviour describing in detail steps for practical implementation.
**Azodo (2011)**	Cross-sectional survey study	Nigeria; five teaching hospitals	138 dental professionals	Prevalence of workplace violence in oral healthcare centres	Survey on prevalence, types, perpetrators, reasons and impact of violence	6.8% of respondents had experienced sexual harassment; reasons and impact of sexual harassment were not distinguished from other forms of violence/harassment
**Baniulyte (2023)**	Cross-sectional survey study	UK; online	214 dental professionals of any level (including students) nationally	Prevalence of inappropriate behaviour, reflect on personal experiences, impact of behaviours on recruitment, progression and dynamics, and improve awareness and accountability	14-item survey on experiences of inappropriate behaviours, perpetrators, and raising concerns to incidents	41% and 39% had experienced or witnessed sexual harassment respectively; 0.7% and 0.1% had experienced or witnessed sexual assault. Perpetrators and reporting mechanisms and outcomes were not clarified for sexual harassment or assault
**Diaz (2022)**	Cross-sectional survey study	United States; online	233 male dental hygienists	Demographics and experiences of men in dental hygiene	41-item survey including demographics,	Total prevalence not clearly stated, though 49 (21%) experienced sexual harassment from patients, 15 (6.4%) from employers,

					professional characteristics and experiences relating to discrimination and harassment	and 23 (9.9%) from coworkers. Sexual harassment by patients was reported by approximately 20% of the participants with less than 10% reporting this was perpetrated by co-workers or employers.
**Ellis (2020)**	Media search	United Kingdom; media outlets	122 media articles	Explore reports on dental professional behaviours in newspaper media	Analysis of newspaper articles in the top 10 UK newspapers	14 articles reported on sexual misconduct, with dramatization and sensationalism, not reflective of fitness to practice cases managed by the General Dental Council
**Foong-Reichert (2023)**	Disciplinary action case analysis	Canada; dental regulatory bodies in 5 provinces	344 disciplinary cases	Reasons for disciplinary action, consequences, and associations with demographic factors for dentists	Coding of publicly available disciplinary action cases from regulatory body websites	Sexual misconduct was not a common reason for disciplinary action (more often clinical incompetence or professional misconduct). Transparency in disciplinary processes is critical for public trust and professional accountability.
**Gallagher (2021)**	Deductive thematic analysis of case data	United Kingdom; professional regulatory bodies	18 relevant disciplinary cases (of 344 total)	Examine the fitness-to-practice process for UK health professionals involved in sexual misconduct and the rationale for sanctions	Document analysis of publicly available case determinations relating to sexual misconduct	Most cases resulted in sanctions, including erasure, regardless of direct patient safety risk. 'Erasure is necessary to maintain public and professional trust, even absent direct patient safety risks.' Regulators emphasize professional standards and public confidence over case-by-case risk evaluations.
**Garbin (2010)**	Cross-sectional survey study	Brazil; single university dental school	208 dental students	Sexual harassment prevalence, and experiences and attitudes of undergraduate dental students	Self-administered 18-item survey during a scheduled class; 11 questions on personal experiences or witnessing of	15% had been sexually harassed (~1/3 due to male patients, 1/3 female patients, 1/3 male professors), of these 19 (9.1%; 8 women and 11 men) participants had been sexually harassed by patients. In addition, 53 (25.4%) had witnessed a student colleague being sexually harassed (about half from patients and half from

					sexual harassment	faculty). Witnesses of harassment believed that most instances were partially the fault of the victim due to 'own inappropriate behaviour or a close relationship with the patient'. ~40% reported they wouldn't know what to do if involved in an incident.
**Ghoneim (2022)**	Cross-sectional survey study	Canada; online	3780 dental hygienists	Experiences of different forms of mistreatment for dental hygienists in dental workplaces	A 72-item self-administered survey; demographics, relevant training, workplace policies, experiences of mistreatment, and reasons for inaction in reporting	23.9% and 15.7% had experienced or witnessed sexual harassment respectively; 2.7% and 2.3% had experienced or witnessed sexual assault respectively. Perpetrators were most commonly patients followed by dentists. Respondents reported either not having (42%) or were unaware (18%) of formal policies for harassment. 42%, 36%, and 37% reported experiencing mistreatment from dentists, office managers, and coworkers respectively.
**Heaton (2020)**	Cross-sectional survey study	United States; American Assoc. for Dental Research conferences	824 attendees of the 2015-2018 conferences	Assess perceptions and experiences of sexual harassment, associations of characteristics, and collect facilitators and solutions to address harassment	Self-administered survey; demographics, experiences of sexual harassment and other misconduct	21% experienced one or more types of harassment; gender harassment was more common than sexual harassment. Within this, 8% experienced sexual remarks, 7% ogling, 1% suggestive materials, 2% unwanted advances, and 5% inappropriate touching.
**Hunt (2020)**	Cross-sectional survey study	Unites States; online	161 dental hygienists in Virginia	Prevalence of sexual harassment for dental hygienists	Used the 'Sexual Experienced Questionnaire' (SEQ-W); has 17 situational items under gender	27% reported at least one experience of sexual harassment in the previous 24 months. 27.3% reported gender harassment, 18.6% unwanted sexual attention, and 6.8% sexual coercion. The most common types were being told

					harassment, unwanted sexual attention and sexual coercion.	offensive sexual jokes or stories (21%) and hearing crude and offensive sexual remarks (18%).
**Inglehart (2024)**	Cross-sectional survey study	United States	212 surgeons; paediatric dentistry, prosthodontics, OMFS	Compare experiences of sexual harassment, discrimination of women, stress, and career satisfaction	Survey; demographics, training, observed treatment of women, experiences of harassment and job satisfaction.	Did not report prevalence, rather the average number of incidences experienced. OMFS and Prosthodontics had the highest frequency, with staff, faculty and patients all the most common perpetrators, with other residents being the least common perpetrators.
**Ivanoff et al (2018)**	Cross-sectional survey study	United States, Bulgaria, Brazil, India; one dental school form each country	990 female dental students	Assess and compare female dental student perceptions of gender bias and experiences of sexual misconduct	24-item self-administered survey distributed at each school, including perceived bias against female students, experiences of abuse or assault	Participants from the US, Brazil, Bulgaria, and India reported that 6%, 6.2%, 2.5%, and 0% respectively reported experiencing some form of sexual assault. The study suggests that gender and cultural differences impact perceptions and experiences of sexual misconduct in educational settings.
**Kim (2017)**	Cross-sectional survey study	South Korea; dental hospitals and clinics in one region	224 dental hygienists	Investigate workplace bullying and sexual harassment and explore countermeasures and preventive measures	46-item self-administered questionnaire survey; demographics, workplace bullying and experience of sexual harassment	Nearly half (48.7%) reported experiencing sexual harassment, with abusive language and leering/staring most common. Visual and physical harassment were less common. Reference to the close physical proximity of dental hygienists to dentists as a potential risk factor; 67.3% of harassment offenders were dentists. 84.5% did not report or confront the perpetrator.

**Liaw (2022)**	Cross-sectional survey study	New Zealand; single university dental school	185 dental students	Prevalence and impact of harassment by patients on dental students	14-item self-administered survey; demographics, number and types of harassment	9 (4.9%) participants (all female) experienced sexual harassment; unwanted compliments/sexual remarks and touching were most common. "He made very inappropriate sexual jokes about dental dams being like condoms, (and) made a comment saying 'it is interesting having a young girl put her fingers in your mouth'."
**Llewellyn (2016)**	Restorative Justice Process Analysis	Canada; single university dental school	29 fourth-year dental students	Address harms caused by misconduct within the dental student cohort, and evaluate culture and climate	Interviews, workshops, group discussions, and restorative process documentation	Not quantified; focussed on qualitative impacts which are comprehensively detailed in participants' statements in the report. Highlighted systemic issues within the Faculty's culture and climate.
**Millbank (2020)**	Review of publicly available tribunal data	Australia; tribunal data	794 disciplinary cases	To examine if uniform law produces consistent outcomes between five professions and jurisdictions	Review of public tribunal data; respondent attributes, type of allegation, and outcomes including severity	Men comprised 80.4% of respondents in sexual misconduct matters, and 96.7% for inappropriate sexual contact were male. Dentists comprised 6.5% of dataset, but were overrepresented relative to their profession (double workforce proportion)
**Patel (2021)**	Cross-sectional survey study	United States; online	232 dental hygienists	Experiences of inappropriate sexual behaviour (IPSB) in the workplace perpetrated by patients	71-item survey, including 6 demographic and 65 relating to IPSB risk and experience	Career occurrence of IFSB of 85.8%; 82.3% patients staring at body parts, 85.8% sexually suggesting remark, 53% asking for a date, 9.1% sexual/romantic gift, 72.4% sexual joke, 12.1% proposed a sexual activity, 41.3% sexually suggesting gestures, 7.3% deliberately exposed genitals or breasts, 6.5% patient masturbated during dental session, 24.1% were purposefully touched or grabbed in a sexual manner, 14.3% were followed,

						watched or harassed inside or outside workplace. "Most patients with inappropriate sexual behaviours were men over 60." There was a reported lack of support by supervisors/senior colleagues, through being dismissed or ignored.
**Premadasa (2011)**	Cross-sectional survey study	Sri Lanka; single university dental school	65 dental students	Perceived mistreatment of student students, measures taken by students and negative consequences of abuse	80-item anonymous self-administered survey during a scheduled class	18% experienced sexual harassment, mostly being stared/leered at or unwelcome sexual comments or jokes. There were no instances of sexual advances involving physical contact or unwanted touching. Fellow male students were the most common perpetrators.
**Quick (2014)**	Mixed methods (survey and focus groups)	United States; single university dental school	188 dental students	Perceptions of factors affecting the dental school environment, and experiences relating to their gender	Structured focus group (interpersonal challenges, effects of challenges, how to manage) and survey	Had a broader focus on male and female differences in dental school, of which harassment was a small part. Experiences of sexual harassment were 34% for women and 7% for men, mostly sexual slurs and advances.
**Rostami (2010)**	Cross-sectional survey study	United States; online	216 female dental oral & maxillofacial surgeons	Tabulate personal and professional characteristics of female dental residents and practitioners	Self-administered survey; demographics, practice characteristics, experiencing biases or sexual harassment; satisfaction with speciality	29% of residents and 38% of practitioners reported experiencing sexual harassment during their careers. Caucasians (80%) were more likely to claim sexual harassment. "OMFS continues to be an 'old boys club,' even in 2008." Despite facing bias, most respondents (93% of residents) would recommend OMFS to other women, citing determination and resilience as key to success.

**Shakeel (2022)**	Cross-sectional survey study	Pakistan; dental institutions and clinics	524 dental surgeons	Awareness and experiences of violence and aggression, including reporting and prevention approaches	Self-administered survey; demographics, experiences of abuse or violence, perceptions of laws and policies on abuse	Broader focus on violence and abuse, minimal information present about sexual harassment. 6.9% had experienced sexual abuse; 78.4% of perpetrators were patients or their relatives.
**Won (2021)**	Cross-sectional survey study	Korea; dental clinics, hospitals and university hospitals	201 dental hygienists	Develop, validate and use a verified tool to measure experiences of workplace violence	31-item self-administered survey; five domains; verbal attacks and alienation, inappropriate work experiences physical threats, sexual harassment, verbal violence	Not possible to accurately determine the total prevalence, though from the 6 statements on sexual harassment, 19 (9.5%) had a coworker physically touch them, 22 (10.9%) had a coworker make an obscene joke or story, 36 (17.9%) had a coworker make sexual innuendos or comment on appearance, 16 (8.0%) had a coworker ask for sexual information of distributed information of a sexual nature, 23 (11.4%) had a coworker gaze unpleasantly, and 15 (7.5%) had a coworker use language or behaviour that induced sexual humiliation. In addition, 15 (7.5% had a patient say something sexually insulting.
